# Serum Soluble ST2 Is a Valuable Prognostic Biomarker in Patients With Acute Heart Failure

**DOI:** 10.3389/fcvm.2022.812654

**Published:** 2022-02-09

**Authors:** Zeyu Wang, Xin Pan, Hong Xu, You Wu, Xiaomin Jia, Yiling Fang, Yi Lu, Yawei Xu, Ji Zhang, Yang Su

**Affiliations:** ^1^Department of Cardiology, Shanghai Tenth People's Hospital, School of Medicine, Tongji University, Shanghai, China; ^2^Geriatric Department, Shanghai Tenth People's Hospital, School of Medicine, Tongji University, Shanghai, China; ^3^Department of Cardiology, The Sixth People's Hospital of Nantong, Nantong, China

**Keywords:** soluble suppression of tumorigenicity 2 (sST2), acute heart failure, cardiovascular death, prognosis, biomarker

## Abstract

**Background:**

This study aimed to investigate the clinical utility of different soluble suppression of tumorigenicity 2 (sST2) levels in assessing the severity and prognosis of patients with acute heart failure (AHF).

**Methods:**

This was a prospective cohort study. Three hundred and thirty-one consecutively enrolled AHF patients from March 2018 to November 2019 were divided into 3 subgroups according to sST2 levels: T1 (1.15–7.70 ng/ml; *N* = 110), T2 (7.71–17.24 ng/ml; *N* = 111), and T3 (17.26–47.42 ng/ml; *N* = 110). The patients were followed up for a median period of 21.0 months for the development of the primary endpoint. Cox proportional hazards model was performed to evaluate the prognostic value of sST2 for the clinical outcomes.

**Results:**

The mean age of patients was 69 years (range, 34–93 years), and 70.4% were male. During the follow-up period, 63 participants died. Patients with higher sST2 levels had lower left ventricular ejection fraction (correlation = −0.119, *P* = 0.031), and higher New York Heart Association classification (correlation = 0.443, *P* < 0.001) and N-terminal pro-B type natriuretic peptide (NT-proBNP) levels (correlation = 0.392, *P* < 0.001). Higher sST2 was also associated with creatinine, urea nitrogen, hemoglobin, and left ventricular mass index. Multivariate analysis revealed that sST2 (per log unit, hazard ratio: 2.174, 95% confidence interval [CI] 1.012–4.67, *P* = 0.047) and NT-proBNP (per log unit, HR 2.171, 95%CI 1.169–4.032, *P* < 0.001) were independent risk factors for the primary outcome in all patients with AHF.

**Conclusion:**

sST2 can provide prognostic information in AHF. The higher the sST2 level in patients with AHF, the higher the incidence of cardiovascular death.

## Introduction

Heart failure (HF) is a complex clinical syndrome caused by impaired systolic and diastolic function. The most serious consequence of HF is the development of acute decompensated HF (AHF); this is associated with significantly increased risks of HF recurrence, rehospitalization, progressive myocardial remodeling, or death (1, 2). Assessing severity in patients with AHF is difficult, and this may lead to incorrect risk stratification and treatment.

Therefore, there is an urgent need to identify novel biomarkers to identify high-risk patients (3). At present, natriuretic peptides (e.g., B-type natriuretic peptide, BNP, and its amino-terminal cleavage fragment, NT-proBNP) are the standard biomarkers for HF used in clinical practice (4). Efforts have been made to identify novel biomarkers in addition to BNP or NT-proBNP, with suppression of tumorigenicity 2 (ST2) emerging as the most promising candidate (5).

Soluble ST2 (sST2) is a member of the interleukin (IL) receptor superfamily and participates in a broad range of biological processes relevant to cardiovascular disease (3, 6). sST2 is closely associated with myocardial fibrosis and myocardial remodeling, and has been identified as a reliable prognostic biomarker for chronic HF (CHF) (7).

Nevertheless, the role of sST2 in patients with AHF is controversial (8), especially the threshold of ST2 concentration and the association with AHF prognosis (9, 10). Therefore, this study evaluated the clinical value of different sST2 levels on the severity and prognosis of AHF patients.

## Methods

### Study Population

**Figure 1:**
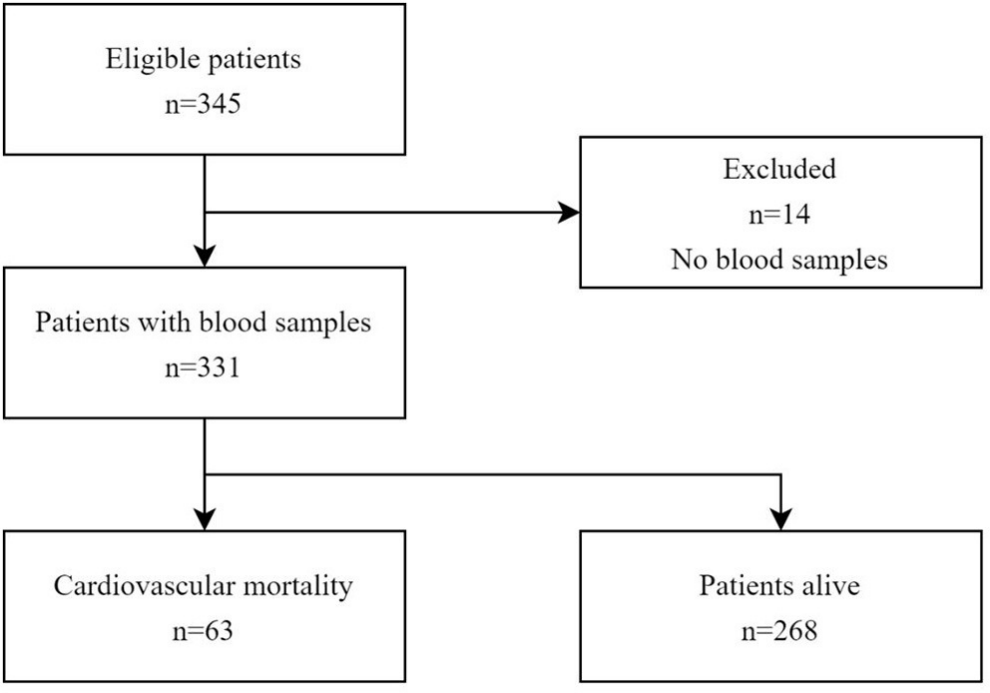
Three hundred and forty-three patients met the eligibility criteria, of whom 331 had blood samples for analysis. All 331 patients were followed up of 21.0 months.

This study was designed as a prospective cohort study. Consecutive patients hospitalized in Shanghai Tenth People's Hospital from March 2018 to November 2019 were enrolled. We included patients over 18 years of age who were admitted with a diagnosis of AHF (11). The exclusion criteria were cardiac diseases including coronary syndrome and myocarditis; severe hepatic, autoimmune, or severe systemic inflammatory diseases; and cancer. Finally, 345 patients were registered (Figure 1). Fourteen patients were excluded due to lack of blood sample data; finally, 331 patients with available blood samples for NT-proBNP and ST2 were included in the current study cohort. The endpoint was cardiovascular death. The data was partially extracted from a registered study (DRAGON-HF) and conformed to the Declaration of Helsinki and its later amendments. The present study has been approved by the local ethics committee of Shanghai Tenth People's Hospital. Written informed consent was acquired from each patient after enrollment.

### Serum Measurement

Blood samples were collected within 12 h after admission. Samples for serum sST2 were centrifuged for 15 min at 3000 r/min and were collected in lyophilized tubes and stored at −80°C. Serum sST2 levels were measured uniformly using enzyme-linked immunosorbent assay (ELISA), and the kit used was purchased from R&D Systems (item number: DY523B-05).

### Collection of Clinical and Echocardiographic Variables

Hospital records were reviewed to collect clinical, analytical, and echocardiographic data. Patients were followed up by telephone calls or clinical visits. The follow-up time was calculated from discharge to cardiovascular death or termination of the study.

### Statistical Analysis

Continuous variables were presented as mean ± standard deviation (SD) or median with interquartile range (IQR) and compared using ANOVA test if they conform normal distribution or Kruskal-Wallis test if not. Categorical variables were expressed as percentages and compared using the chi-square test. Correlations were analyzed with Pearson test or Spearman rank correlation coefficients. Survival curves were estimated and plotted using the Kaplan-Meier method, and the log-rank test was used to compare groups. The association between primary endpoint and variables was presented as hazard ratio (HR) and 95% confidence interval (CI). Because sST2 and NT-proBNP have a skewed distribution, we performed logarithmic conversion on the data in the Cox regression analyses. *P* < 0.05 was considered statistically significant. Statistical analysis was performed with SPSS 20.0.

## Results

### Comparisons of Clinical Characteristics and Echocardiographic Parameters

The mean age of the patients in this study was 69 years, and 70.4% were male. The mean sST2 level of the entire population was 14.42 ± 10.06 ng/ml; all participants were divided into 3 subgroups according to sST2 tertiles (T1: 1.15–7.70 ng/ml; T2: 7.71–17.24 ng/ml; T3: 17.26–47.42 ng/ml). The baseline characteristics are shown in Table 1. Although left ventricular mass index (LVMI) did not differ between groups, further gender subgroup analysis revealed that LVMI was significantly correlated with sST2 in men (*P* < 0.05). Spearman correlation analysis revealed positive correlations of sST2 with NT-proBNP and NYHA class among all patients (*r* = 0.392 and 0.443, respectively; *P* < 0.001), while a negative correlation was found with LVEF values (*r* = −0.119; *P* = 0.031; Table 2).

**Table 1:** Baseline characteristics of all patients with different levels of serum soluble ST2 (sST2).

**Variable**	**sST2 (ng/ml)**			***P*-value**
	**T1 (*N* = 110)**	**T2 (*N* = 111)**	**T3 (*N* = 110)**	
Age (years)	67.84 ± 11.7	68.96 ± 10.84	69.95 ± 12.26	0.376
Gender (male, %)	80 (72.7)	79 (71.2)	74 (67.3)	0.659
Smoker, yes (%)	36 (32.7)	41 (36.9)	25 (22.7)	0.063
SBP (mmHg)	137.98 ± 25.52	137.93 ± 23.58	133.80 ± 22.25	0.358
DBP (mmHg)	79.42 ± 16.55	79.66 ± 15.50	79.65 ± 16.88	0.939
BMI (kg/m^2^)	24.77 ± 3.42	24.08 ± 3.13	24.17 ± 3.98	0.181
Past medical history				
Atrial fibrillation (%)	26 (23.6)	22 (19.8)	28 (25.5)	0.596
Coronary heart disease (%)	83 (75.5)	94 (84.7)	81 (73.6)	0.104
Diabetes mellitus (%)	41 (37.3)	42 (37.8)	35 (31.8)	0.588
Hypertension (%)	74 (67.3)	74 (66.7)	77 (70.0)	0.852
NYHA functional class (%)				*P* < 0.001
II	80 (51.3)	45 (28.8)	31 (19.9)	
III	23 (20.9)	45 (40.9)	42 (38.2)	
IV	7 (10.8)	21 (32.3)	37 (56.9)	
Laboratory findings				
Hemoglobin (g/L)	134.99	129.99	126.11	*P* < 0.001
	(127.00–145.25)	(119.99–139.00)	(111.10–140.00)	
C-reactive protein (mg/L)	6.15	4.19	6.91	0.806
	(3.02–18.97)	(3.02–17.55)	(3.02–19.61)	
ALT (U/L)	23.65	24.31	25.01	0.533
	(15.12–37.35)	(15.21–44.07)	(14.80–34.91)	
Creatinine (umol/L)	85.65	85.55	96.91	0.020
	(72.55–107.75)	(71.32–110.22)	(77.31–128.21)	
Urea nitrogen (umol/L)	7.05	6.45	8.61	*P* < 0.001
	(5.44–9.12)	(5.30–8.93)	(6.21–11.10)	
LDLC (mmol/L)	2.11 ± 0.90	2.17 ± 0.98	2.11 ± 0.90	0.894
HDLC (mmol/L)	0.98 ± 0.33	1.00 ± 0.29	0.98 ± 0.33	0.823
HBA1C	7.01 ± 1.62	6.68 ± 1.58	6.75 ± 1.54	0.404
K (mmol/L)	4.03 ± 0.58	4.06 ± 0.50	4.00 ± 0.61	0.827
Na (mmol/L)	140.98 ± 3.34	141.15 ± 3.78	140.73 ± 3.99	0.604
HS-TNT (ng/ml)	0.03	0.05	0.06	0.024
	(0.01–0.11)	(0.01–0.37)	(0.02–0.27)	
NT-proBNP (pg/ml)	1012.5	2170.0	3678.0	*P* < 0.001
	(483.8–2424.5)	(1023.0–5597.0)	(1875.2–8616.0)	
Echocardiography parameters				
LVEF	44.63 ± 13.33	44.28 ± 12.95	41.27 ± 13.63	0.040
LVMI (male)	94.23	102.95	113.6	0.042
	(82.30–128.55)	(82.42–135.49)	(90.97–113.28)	
LVMI (female)	94.01	90.51	90.28	0.713
	(82.30–106.05)	(74.43–105.71)	(77.20–109.02)	

*The entire study population was divided into T1, T2, and T3 groups according to the tertile of serum sST2 concentration*.

*BMI, Body Mass Index; LVEF, left ventricular ejection fraction; SBP, systolic blood pressure; DBP, diastolic blood pressure; ALT, alanine aminotransferase; LVMI, left ventricular mass index*.

*Data are expressed as mean + SD or number (%) and median (quartiles)*.

**Table 2:** Correlation of clinical/echocardiographic parameters with sST2.

**sST2**	**Correlation coefficient**	***P*-value**
NT-proBNP	0.392	*P* < 0.001
LVEF	−0.119	*P* = 0.031
NYHA	0.443	*P* < 0.001

*LVEF, left ventricular ejection fraction*.

### Relationship Between sST2 and NYHA Functional Class in AHF Patients

**Figure 2:**
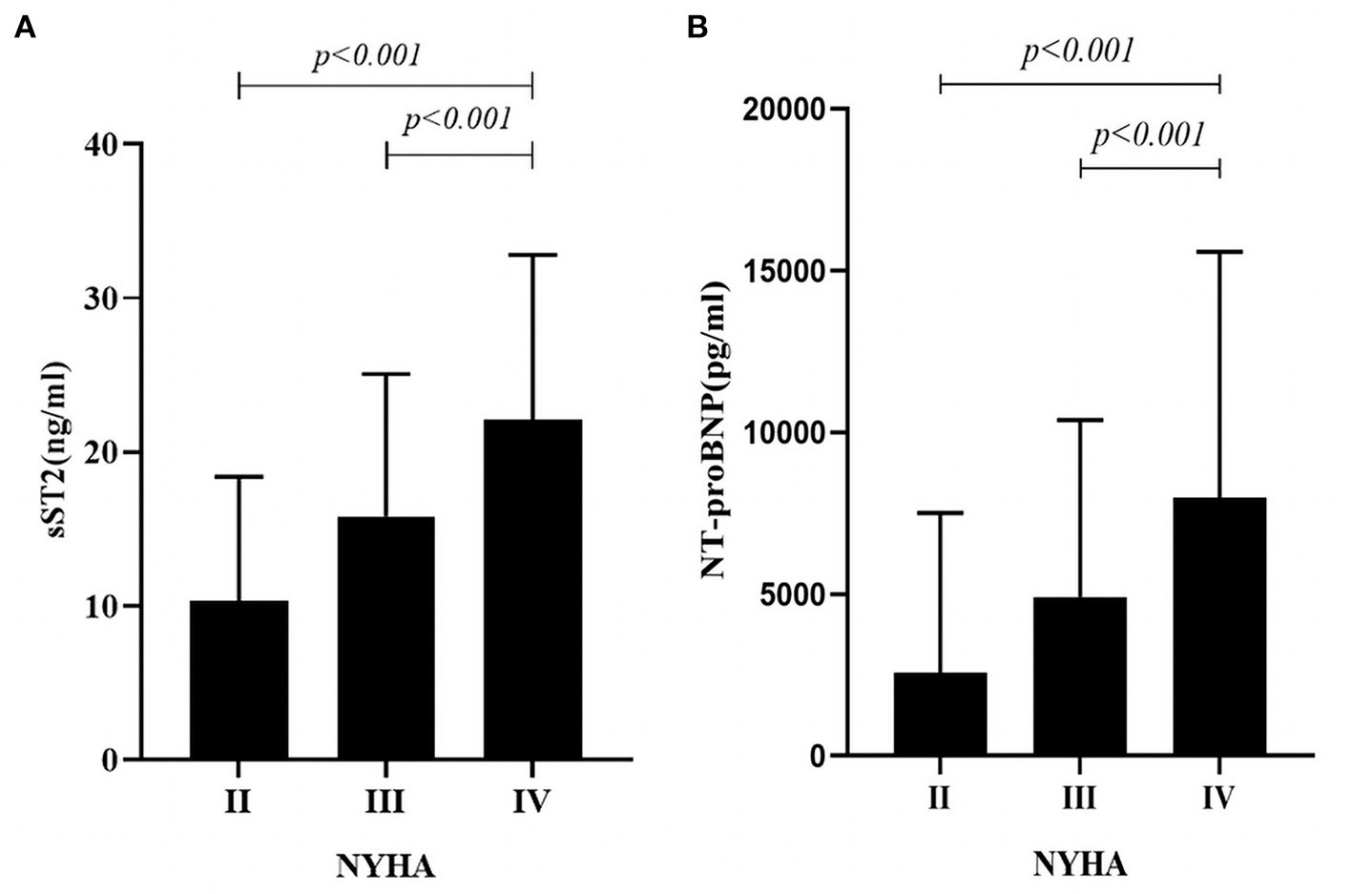
**(A)** Relationship Between sST2 and NYHA Functional Class in AHF Patients. The sST2 concentrations were significantly different between groups and patients with higher NYHA class displayed higher sST2 levels (10.27 ± 8.09 vs. 15.80 ± 9.17 vs. 22.06 ± 10.72; *P* < 0.001; **(B)** Relationship Between NT-proBNP and NYHA Functional Class in AHF Patients. The NT-proBNP concentrations were significantly different between groups and patients with higher NYHA class displayed higher NT-proBNP levels (2577.26 ± 4956.30 vs. 4868.71 ± 5464.38 vs. 7972.41 ± 7601.53; *P* < 0.001).

Participants were divided into 3 subgroups according to NYHA classification; we found that sST2 and NT-proBNP concentrations were significantly different between groups and patients with higher NYHA class displayed higher sST2 levels (10.27 ± 8.09 vs. 15.80 ± 9.17 vs. 22.06 ± 10.72; *P* < 0.001; Figure 2A) and higher NT-proBNP level (2577.26 ± 4956.30 vs. 4868.71 ± 5464.38 vs. 7972.41 ± 7601.53; *P* < 0.001; Figure 2B).

### Relationship Between sST2 and LVEF Levels in AHF Patients

**Figure 3:**
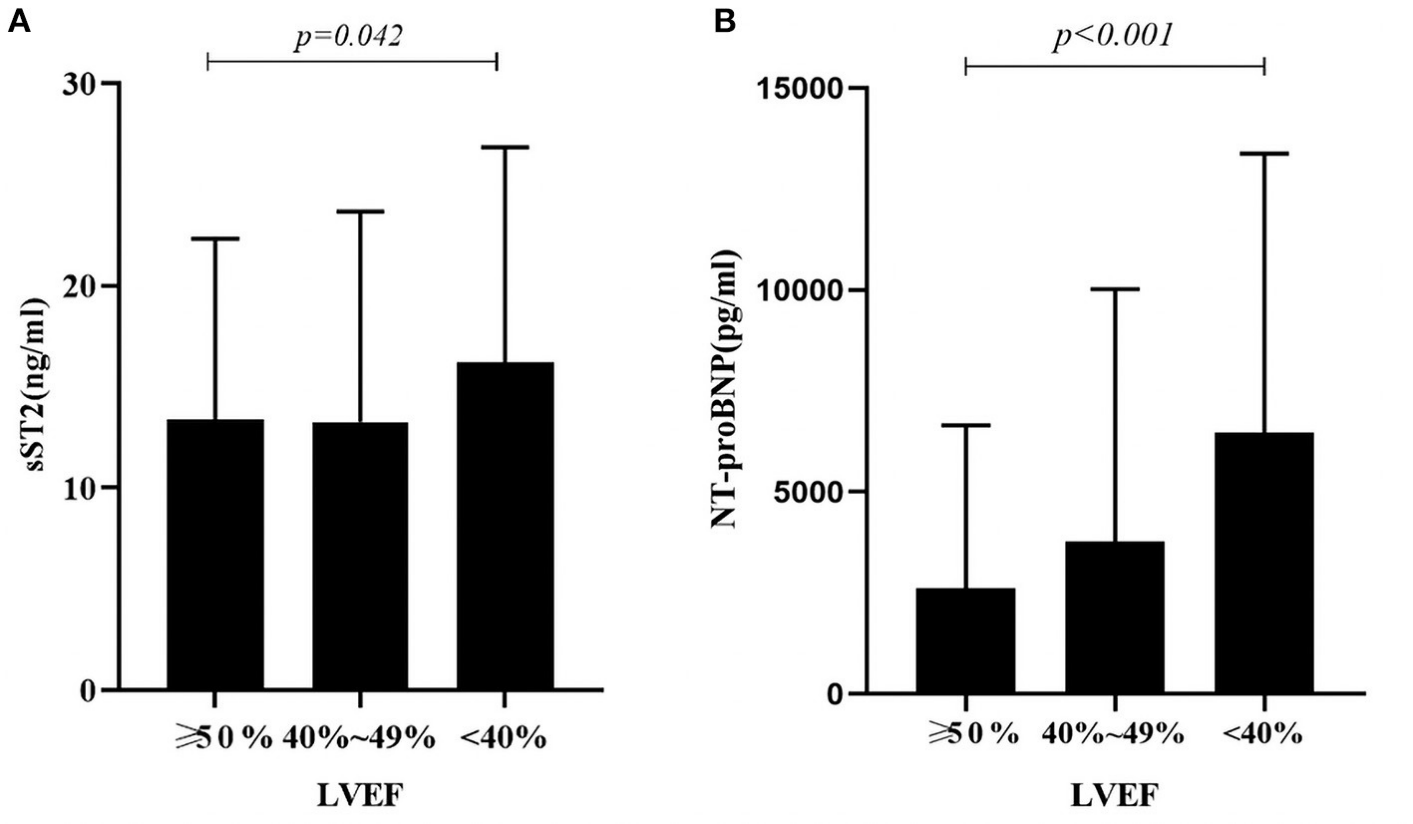
**(A)** Relationship Between sST2 and LVEF Levels in AHF Patients. There was a statistically significant difference in sST2 between the three groups. Patients in the HFrEF group displayed the highest level of sST2 (sST2: 16.21 ± 10.64, *P* = 0.042); **(B)** Relationship Between NT-proBNP and LVEF Levels in AHF Patients. There was a statistically significant difference in NT-proBNP between the three groups. Patients in the HFrEF group displayed the highest level of NT-proBNP (NT-proBNP: 6874.0 ± 6899.5, *P* < 0.001).

Patients were divided into 3 subgroups to compare the association of sST2 and NT-proBNP levels according to different LVEF levels: HF with reduced ejection fraction (HFrEF, left ventricular EF [LVEF] < 40%), HF with mid-range EF (HFmrEF, 40% ≤ LVEF ≤ 49%), and HF with preserved EF (HFpEF, LVEF ≥ 50%). We found statistically significant differences in sST2 and NT-proBNP between the three groups. Patients in the HFrEF group displayed the highest level of sST2 and NT-proBNP (sST2: 16.21 ± 10.64, *P* = 0.042; NT-proBNP: 6874.0 ± 6899.5, *P* < 0.001; Figures 3A,B).

### The Correlation and Prognostic Value of sST2 for Outcome in AHF Patients

**Figure 4:**
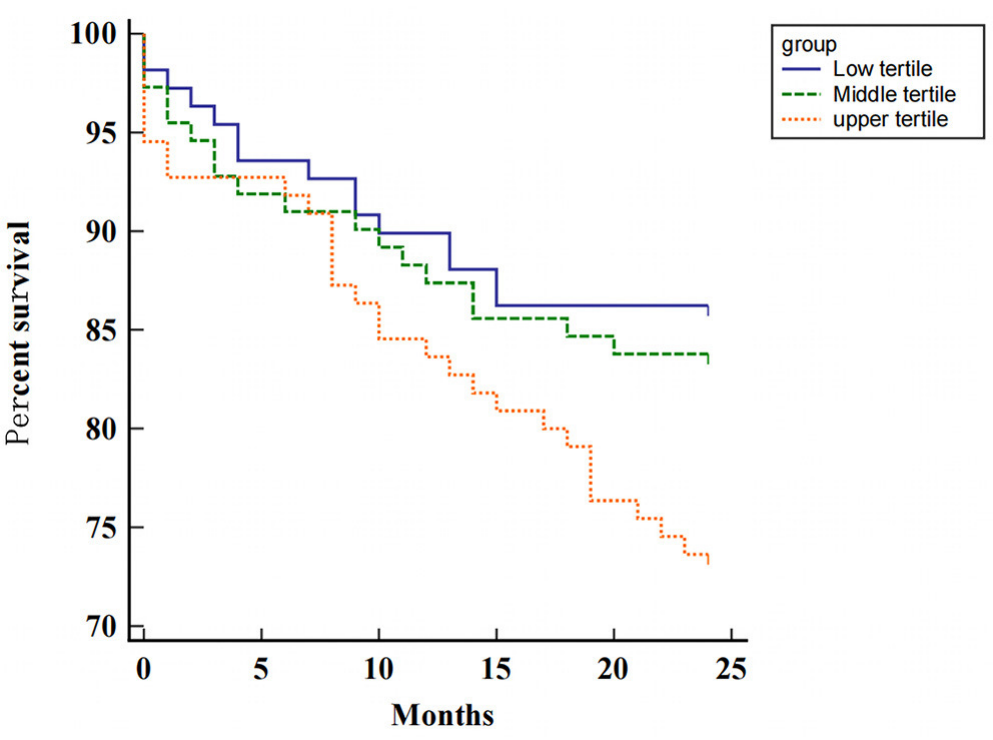
Kaplan-Meier survival curves according to the level of sST2 in all patients.

We assessed the relationship between sST2 levels and patient outcome. Kaplan-Meier curves estimating the event-free survival (cardiovascular mortality) were presented according to different sST2 groups. There were total of 63 cardiovascular deaths over a median follow-up of 21.0 months. Patients in the upper tertile of sST2 had a lower survival rate after discharge than those in the middle and lower tertiles (log-rank test *P* = 0.048; Figure 4). Cox regression analysis for cardiovascular mortality is presented in Table 3. In crude analysis, sST2 (per log unit, HR 2.45, 95% CI 1.172–5.12, *P* = 0.017) and NT-proBNP (per log unit, HR 3.033, 95%CI 1.826–5.037, *P* = 0.014) were significantly associated with the primary outcome. After adjusting for age, sex, body mass index, C-reactive protein, smoking, creatinine, urea nitrogen, and LVMI, the associations of sST2 (per log unit, HR 2.174, 95% CI 1.012–4.67, *P* = 0.047) and NT-proBNP (per log unit, HR 2.171, 95% CI 1.169–4.032, *P* < 0.001) were maintained.

**Table 3:** Crude and adjusted hazard ratios (95% confidence interval) of sST2 and NT-proBNP for the primary outcome.

	**HR (95% CI)**	***P*-value**
ST2		
Crude	2.45 (1.172–5.12)	0.017
Adjusted	2.174 (1.012–4.67)	0.047
NT-proBNP		
Crude	3.033 (1.826–5.037)	0.014
Adjusted	2.171 (1.169–4.032)	*P* < 0.001

*After adjusting for age, sex, BMI, C-reactive protein, smoking, creatinine, urea nitrogen, LVMI*.

## Discussion

This study compared the clinical characteristics and prognosis of patients with AHF and different sST2 levels. We found that sST2 was highly correlated with the severity and poor prognosis of AHF. As HF severity increased and the clinical condition of patients worsened, sST2 levels tended to increase.

AHF is the final battleground of cardiovascular disease and the most stubborn bastion (1). AHF is the leading cause of hospitalization in the elderly, and despite current advances in treatment, it is associated with high morbidity and poor prognosis (12). During hospitalization, patients with AHF have a mortality rate between 4 and 7%; this increases up to 7–11% in the first 2–3 months after discharge, and up to 36% in 1 year (13, 14). It has been shown that the expression levels of various biomarkers in the early stages of HF are strongly associated with the severity and prognosis of HF (15). Furthermore, serum biomarkers may play an essential role in bridging the gap between the assessment of AHF and the occurrence of adverse outcomes.

sST2 is a member of the IL-1 receptor family and is currently well studied in its two isoforms, the soluble form (sST2) and the transmembrane form (ST2L). sST2 is mainly released by vascular endothelial cells and expressed by hematopoietic and epithelial cells that regulate THf2-type responses. IL-33 is a functional ligand for sST2 and ST2L, and the beneficial effects of IL-33 are transmitted through ST2L to form IL-33/ST2L, which exerts a protective effect against over-response. The IL-33/ST2L signaling pathway is a mechanically activated system that inhibits cardiac hypertrophy and fibrosis (16). Acute decompensated HF occurs when the ventricular volume load increases dramatically in a short period, and cardiomyocytes and fibroblasts secrete excessive sST2 and ST2L in response to stress stimulation. The elevated sST2 partially replaces ST2L and IL-33 binding, forming IL-33/sST2, and this combined ligand does not have a protective role on the heart (5).

This study shows that LVMI increased with ST2 levels, reflecting that ST2 is a reliable indicator of myocardial hypertrophy, which is in line with the findings of Chen et al. (17), Veeraveedu et al. (18), Ojji et al. (19), and Sanada et al. (20) previously also confirmed the relationship between serum ST2 levels and hypertrophic myocardium, and demonstrated that the IL-33/ST2L system has a biomechanical activation effect which can regulate cardiomyocyte hypertrophy. Farcaş et al. (21) found that ST2 can be used as a diagnostic biomarker for myocardial hypertrophy. These results indicate that ST2 plays a role in the occurrence of myocardial hypertrophy. IL-33 produced by cardiac fibroblasts significantly antagonizes angiotensin II and phenylephrine and inhibits cardiomyocyte hypertrophy. sST2 blocks the action of IL-33, which leads to myocardial hypertrophy. Therefore, the conclusion of this study is consistent with the above-mentioned studies showing that ST2 is a reliable indicator of myocardial hypertrophy.

We found that the expression levels of sST2 and NT-proBNP can be used as an important indicator of the severity of patients with AHF because their expression levels increased with the aggravation of NYHA functional class. Rehman et al. (22) studied the link between sST2 levels and clinical parameters. They found that ST2 and NT-proBNP are associated with NYHA cardiac function. Immanuel et al. (23) showed that sST2 levels are expressed at higher levels with worsening cardiac function. All these studies confirmed the validity of the current results.

By comparing sST2 and NT-proBNP levels in HF patients with different LVEF levels, we found that sST2 and NT-proBNP levels were significantly higher in HFrEF than in patients with HFmrEF and HFpEF. Pan et al. (24) and Sanders-van et al. (25) observed the relationship between NT-proBNP and LVEF in HF, and found that HFrEF patients have a greater plasma NT-proBNP than HFpEF and HFmrEF patients. Huang et al. (26) found sST2 was higher in HFrEF than in patients with HFmrEF and HFpEF. Our findings are consistent with the above studies. However, some scholars have different views on the concentration of ST2 in patients with different LVEF levels. Friões et al. (27) found no difference in the concentration of sST2 according to LVEF category in HF patients, while Song et al. (28) revealed sST2 was higher in HFpEF than in patients with HFmrEF and HFrEF. A possible reason for these controversial results, also supported by Huang et al. (26) could be due to the research population. As demonstrated by Tseng et al. (29) the level of sST2 in patients with end-stage HF increased sharply, but it decreased within 3 months after implantation of the left ventricular assist device. In our HFrEF group, some patients with end-stage HF had severe AHF with dyspnea and lower limb edema, which may have caused the patients' sST2 levels to rise abruptly and remain extremely high over time.

This study also found that higher baseline sST2 levels are associated with increased risk of cardiovascular death, and ST2 is an influential and independent predictive factor of adverse prognosis in patients with AHF. The PRIDE study (30) proved that patients with increased levels of sST2 had the highest risk of death at 1 year (>40%), and in the multivariate Cox regression analysis of independent predictors of death, sST2 was related to mortality. Januzzi et al. (3) suggested that high sST2 levels may be used to reclassify patients at a higher risk of mortality, indicating that sST2 dramatically raises traditional risk stratification markers in patients with AHF. Other studies have reported similar results (28, 31, 32). Our study concluded that high ST2 concentration (>17.26 ng/ml) had a stronger correlation with adverse prognosis in AHF.

Furthermore, we found sST2 predicts end-point events better than NT-proBNP in multivariate cox regression, and these results are consistent with those of Pan et al. (24). A possible reason for this is that unlike NT-proBNP, sST2 is not affected by body mass index, renal function, or age (33).

This study has some limitations worth noting. The sample size of this study is small, and its findings need to be further confirmed by a large sample and multicenter study. Further, the study was also limited by the lack of continuous sST2 data. Nevertheless, our results are valuable and may be used in future studies in combination with other HF biomarkers to improve the assessment of HF patients.

In conclusion, sST2 is highly correlated with the LVEF and the NYHA class in patients with AHF, suggesting its high clinical value in assessing the severity of HF. sST2 can provide prognostic information in AHF. Higher sST2 level in patients with AHF is related to a higher incidence of adverse events.

## Data Availability Statement

The raw data supporting the conclusions of this article will be made available by the authors, without undue reservation.

## Ethics Statement

The studies involving human participants were reviewed and approved by the data was partially extracted from a registered study on the web of Clinical Trial Registry (NCT03727828, DRAGON-HF trail) and conformed to the Declaration of Helsinki and was approved by the Ethics Committee of Shanghai Tenth People's Hospital. The patients/participants provided their written informed consent to participate in this study.

## Author Contributions

Conception, study design, and administrative support: YS and JZ. Provision of study materials or patients and Final approval of manuscript: ZW, XP, HX, YW, XJ, YF, YL, YX, JZ, and YS. Collection and assembly of data: ZW, XP, HX, and YS. Data analysis and interpretation: ZW, XP, and JZ. Manuscript writing: ZW and YS. All authors contributed to the article and approved the submitted version.

## Conflict of Interest

The authors declare that the research was conducted in the absence of any commercial or financial relationships that could be construed as a potential conflict of interest.

## Publisher's Note

All claims expressed in this article are solely those of the authors and do not necessarily represent those of their affiliated organizations, or those of the publisher, the editors and the reviewers. Any product that may be evaluated in this article, or claim that may be made by its manufacturer, is not guaranteed or endorsed by the publisher.

## Abbreviations

sST2: serum soluble Suppression of Tumorigenicity 2
AHF: acute heart failure
LVEF: left ventricular ejection fraction
LVMI: left ventricular mass index.
